# Congenital leptin deficiency and thyroid function

**DOI:** 10.1186/1756-6614-2-11

**Published:** 2009-11-04

**Authors:** Gilberto Paz-Filho, Tuncay Delibasi, Halil K Erol, Ma-Li Wong, Julio Licinio

**Affiliations:** 1 The John Curtin School of Medical Research, The Australian National University, Canberra ACT, Australia; 2 Ankara Numune Research and Training Hospital, Endocrinology and Metabolism Clinic, Ankara, Turkey; 3 Center on Pharmacogenomics, Department of Psychiatry and Behavioral Sciences, University of Miami Miller School of Medicine, Miami, FL, USA

## Abstract

Thyroid function is closely related to leptin's secretion by the adipose tissue. In states of leptin-deficiency, the circadian rhythm of TSH is altered, leading to central hypothyroidism in animal models. In humans, central hypothyroidism has also been described in rare cases of congenital leptin deficiency. However, the thyroid phenotype in these cases is heterogeneous, with the occurrence of central hypothyroidism in a minority of cases. Here we describe thyroid function in four leptin-deficient humans (2 males aged 5 and 27, and 2 females aged 35 and 40), before and during leptin replacement with recombinant human methionyl leptin (r-metHuLeptin). The child was evaluated for four years, and the adults, for eight years. In addition, the adults were submitted to a brief withdrawal of leptin during six weeks in the sixth year. Our results show that, regardless of leptin replacement, our leptin-deficient patients have normal thyroid function. In spite of having an important role in regulating the hypothalamic-pituitary-thyroidal axis, leptin is not required for normal thyroid function.

ClinicalTrials.gov Identifiers: NCT00659828 and NCT00657605

## Findings

There is evidence that the hypothalamic-pituitary-thyroidal axis is regulated, at least in part, by leptin. This provides an important interface between adiposity, regulated by leptin, and metabolic rate, regulated by thyroid hormones. The mechanisms underlying the connection between adipose signals and energy expenditure include the regulation of the synthesis and secretion of TRH (thyrotropin releasing hormone) by leptin, through the mediation of input from the arcuate nucleus to the TRH neurons in the paraventricular nucleus (PVN) [1]. In addition, the thyroid axis is also indirectly regulated by leptin's actions on the melanocortin pathway, as alpha-MSH (melanocyte stimulating hormone) stimulates and AgRP (agouti-related protein) blocks TRH release [2]. Furthermore, leptin has direct effects on TRH neurons, regulating its synthesis not only by up-regulating the expression of the proTRH gene in the PVN [3] and by influencing the feedback regulation of the TRH-secreting neurons by thyroid hormones, but also by increasing promoter activities of the prohormone convertases PC1/3 and PC2, essential for the activation of TRH from proTRH [4].

In leptin-deficient humans, different thyroid phenotypes have been reported. In three children of Pakistani origin, thyroid function tests were within the normal range before the start of recombinant human methionyl leptin (r-metHuLeptin) therapy, with a rise in free T4 (fT4) thereafter in all children, and an increase in T3 in the two youngest [5]. In another child, also of Pakistani origin, subclinical hypothyroidism was diagnosed before treatment, with high TSH and normal T4 levels [6]. Treatment with levothyroxine (LT4) was initiated before r-metHuLeptin, with a decrease in TSH levels. After the initiation of r-metHuLeptin, fT4 levels increased above the upper limit of the reference range, leading to the interruption of LT4 therapy. While on r-metHuLeptin, off LT4, thyroid function and TSH response to TRH were normal, which gives further support to the hypothesis that the hypothalamic-pituitary-thyroidal is regulated by leptin.

We would like to document here that the phenotype of leptin-deficient patients is highly heterogeneous, by reporting the findings on thyroid function in leptin-deficient patients of Turkish origin. We have been studying three adults (1 male and 2 females) and one boy from a highly consanguineous Turkish family. Those patients have a nonconservative missense leptin gene mutation (cysteine-to-threonine in codon 105), which renders them leptin-deficient. We previously described the effects of treatment with r-metHuLeptin, started at ages 5 (male patient A) [7], 27 (male patient B), 35 and 40 (female patients C and D, respectively) [8].

As opposed to the findings in one child of Pakistani origin, our leptin-deficient boy (A) had normal thyroid function before, two and four years after the initiation of r-metHuLeptin. In addition, we have not observed an increase in fT4 or T3 levels after the initiation of r-metHuLeptin (Table 1).

**Table 1:** Tests of thyroid function in leptin-deficient adults, before and after treatment with r-metHuLeptin.

		**Before leptin**	**One year after**	**Two years after**	**Four years after**	**Six years after**	**Six years after (off leptin)**	**Seven years after**	**Eight years after**
TSH (mU/L)	A	3.8	*	4.5	4.17	*	*	*	*
	
	B	0.93	1.1	*	0.64	1.68	1.26	0.68	1.38
	
	C	1.5	*	*	*	1.09	1.61	1.27	1.0
	
	D	2.4	0.88	*	0.47	0.83	1.15	1.52	1.23

Total T4 (μg/dl)	A	5.4	*	8.8	*	*	*	*	*
	
	B	6.9	6.9	*	9.1	7.5	7.6	*	*
	
	C	8.4	*	*	*	8.0	8.4	*	*
	
	D	5.2	7.9	*	10.9	7.5	6.5	*	*

Total T3 (ng/dl)	A	*	*	*	*	*	*	*	*
	
	B	114	112	167	121	121	*	*	*
	
	C	118	*	*	*	112	118	*	*
	
	D	76	125	*	110	122	106	*	*

Free T4 (ng/dl)	A	1.3	*	1.2	1.49	*	*	*	*
	
	B	*	*	*	*	1.0	*	0.9	1.34
	
	C	*	*	*	*	1.1	1.3	0.8	1.47
	
	D	*	*	*	*	1.1	1.0	0.73	1.16

BMI (kg/m^2^)	A	39.6	24.8	23.8	22.6	*	*	*	*
	
	B	51.4	24.5	22.6	23.7	23.3	26.7	25.8	25.4
	
	C	46.7	26.0	26.0	25.0	26.7	29.0	30.3	32.2
	
	D	55.4	35.0	28.0	31.7	33.3	36.2	32.5	36.5

* Not available

Reference ranges: TSH: 0.40-4.0 mU/L; total T4: 4.5-12.5 μg/dl; total T3: 75-178 ng/dl; free T4: 0.7-2.1 ng/dl

Similarly, the leptin-deficient adults also have normal thyroid function, both before and after the initiation of r-metHuLeptin. Six years after the initiation of treatment, a brief withdrawal of leptin during six weeks was undertaken. No significant changes in thyroid hormones were observed (Table 1). In spite of having normal thyroid function, we have previously shown that the absence of leptin disorganizes the circadian rhythm of TSH [9]. In addition, levels of anti-thyroid antibodies were normal in our patients, at all times.

Based on our data we conclude that in spite of having an important role in regulating the hypothalamic-pituitary-thyroidal axis, leptin is not required for normal thyroid function. Why does leptin deficiency cause different thyroid phenotypes? Leptin is truly pleiotropic, with multiple effects that can directly or indirectly affect thyroid function. Therefore, thyroid dysfunction, in a leptin-deficient state, may exist due to diverse combinations of factors that may vary across patients. In addition, age may be an important determinant of thyroid dysfunction, as only the youngest child showed laboratorial alterations related to thyroid function. It is important to note that not only the thyroid phenotype is heterogeneous among leptin-deficient patients. In comparison with the patients of Pakistani origin, our patients had higher body mass index (BMI), but lower insulin levels and insulin resistance index (HOMA-IR), as illustrated in Table 2. This heterogeneity was observed even among the Turkish patients for several parameters. In addition, the therapeutic regimen was slightly different in both groups of patients, regarding the time of administration of leptin (in the morning vs. in the evening for our patients - mimicking leptin's circadian rhythm). Nevertheless, in both protocols, dose was adjusted based on clinical response. That heterogeneous phenotype may be explained by several factors, such as a different concentration of leptin soluble receptor, leading to variations in free leptin levels. So far, the molecular mechanisms by which different mutations in the leptin gene lead to the presence or absence of thyroid dysfunction is unknown.

**Table 2:** Clinical and biochemical parameters of the Pakistani and Turkish leptin-deficient patients, before treatment with r-metHuLeptin.

	**Pakistani children**	Turkish patients*	**Turkish child**
BMI (kg/m^2^)	41.3 ± 5.5	48.3 ± 6.8	39.6
Insulin (μIU/ml)	20.9 ± 11.5	9.3 ± 8.0	21
Glucose (mg/dl)	77.4 ± 11.4	97.2 ± 23.1	79
HOMA-IR	3.6 ± 1.9	2.1 ± 1.5	4.1
Total cholesterol (mg/dl)	181.2 ± 18.1	149.7 ± 29.5	166
HDL-c (mg/dl)	37.3 ± 10.1	32.8 ± 4.2	36
LDL-c (mg/dl)	85.6 ± 33.7	83.5 ± 16.9	87
Triglycerides (mg/dl)	162.2 ± 60.0	166.7 ± 78.1	216

*including the leptin-deficient child

The multidirectional action of leptin in thyroid axis should also be taken into account. Thyroid hormones can stimulate the transcription of leptin gene through adrenergic effects. Although endogenous hyperthyroidism can lead to higher leptin levels, short-term treatment does not change leptinemia [10], neither does the induction of hyperthyroid states in healthy males [11]. Another study showed that hypothyroid patients have lower leptinemia [12]. In our study, since all patients were euthyroid, this multidirectional action does not apply.

In conclusion, we show here that thyroid dysfunction is not a constant in leptin deficiency. The identification of factors that lead to hypothyroidism in some, but not all, leptin-deficient patients will provide better understanding of the roles of leptin on the hypothalamic-pituitary-thyroidal axis. New insight on the interaction between leptin and thyroid function will be gained from future studies aimed at identifying the factors that protect our leptin-deficient patients from clinical thyroid dysfunction, even in the presence of an abnormal circadian rhythm of TSH.

## Abbreviations

BMI: body mass index; fT4: free T4; HDL-c: high-density lipoprotein cholesterol; HOMA-IR: homeostasis model of assessment of insulin resistance; LDL-c: low-density lipoprotein cholesterol; LT4: levothyroxine; MSH: melanocyte stimulating hormone; PVN: paraventricular nucleus; r-metHuLeptin: recombinant human methionyl leptin; TRH: thyrotropin releasing hormone.

## Competing interests

The authors declare that they have no competing interests.

## Authors' contributions

All authors contributed equally to conception and design, acquisition of data, analysis and interpretation of data; manuscript drafting and final approval was also done by all authors.
